# Executive Function Profiles in Children with Special Educational Needs: A Transdiagnostic Approach

**DOI:** 10.3390/brainsci16101023

**Published:** 2026-09-25

**Authors:** Camila Martínez, Felipe Torres-Morales, Patricia Salazar, Camila Cachaña

**Affiliations:** 1 Center for Advanced Studies on Educational Justice, Pontificia Universidad Católica de Chile, Santiago 7820436, Chile; 2 Departamento de Fonoaudiología, Facultad de Medicina, Universidad de Chile, Santiago 8380453, Chile; feltorres@uchile.cl; 3 Facultad de Educación, Universidad Diego Portales, Santiago 8370067, Chile; patricia.salazar@udp.cl; 4 Centro de Desarrollo de Tecnologías de Inclusión (CEDETi UC), Pontificia Universidad Católica de Chile, Santiago 7820436, Chile; camila.cachana@cedeti.cl

**Keywords:** executive functions, special educational needs, transdiagnostic approach, latent profile analysis, cognitive profiles

## Abstract

**Highlights:**

**What are the main findings?**

- Four different latent profiles emerged from the studied SENs: Specific Inhibition Deficit; Severe Executive Deficit; Mild Executive Deficit; Average Executive Performance.
- A high proportion of children with intellectual disability were classified within the Severe Executive Deficit group.

**What are the implications of the main findings?**

- Executive profiles shared by participants with different SENs suggest the need for a transdiagnostic approach when addressing children’s executive needs.
- Some conditions, such as ADHD and ASD, may require indirect assessments to better describe their executive needs.

**Abstract:**

**Background/Objectives**: Executive Functions are high-order cognitive processes, fundamental for children’s development, often shown diminished in children with Special Educational Needs (SEN). Although literature is consistent in finding these difficulties in children with SEN, the high individual heterogeneity translates into different results within conditions. The present exploratory study addresses this heterogeneity, by using a transdiagnostic person-centered approach, to identify executive profiles across different conditions and to link them to traditional diagnostic labels. Additionally, the relationship between those profiles and sociodemographic characteristics, such as age and gender was explored. **Method**: A total of 185 children (mean age 9.8; SD = 2.61) with diverse SEN diagnoses were assessed: Attention Deficit Hyperactivity Disorder (*n* = 64; mean age 10.92; SD = 2.16), Intellectual disabilities (*n* = 23; mean age 12.65; SD = 1.37), Developmental Language Disorder (*n* = 64; mean age 7.28; SD = 1.25), Learning disabilities (*n* = 13; mean age 10.38; SD = 2.06), and Autism Spectrum Disorder (*n* = 21; mean age 10.57; SD = 1.96). Participants were tested with the Yellow-Red battery test, measuring cognitive and behavioural inhibition; verbal and non-verbal working memory; and cognitive flexibility. Data were analysed by applying Latent Profile analyses. **Results**: Four different profiles emerged: Specific Inhibition Deficit; Severe Executive Deficit; Mild Executive Deficit; and Average Executive Performance. Results showed a high heterogeneity within and a large overlap between the different conditions. Nevertheless, some conditions constituted a high proportion of certain profiles. For instance, Intellectual disabilities accounted for 70% of the Severe Executive Deficit profile. Although age showed a significant relationship to profile membership, gender did not. **Conclusions**: Executive Functions operate as cognitive processes that cross multiple diagnoses, rather than as markers for specific conditions. The results highlight the relevance of focusing on individual cognitive profiles, rather than traditional labels and diagnoses, whenever designing and implementing educational interventions.

## 1. Introduction

Executive functions (EFs) correspond to high-level cognitive processes involved in the control and regulation of an individual’s thoughts and actions. According to the model by Miyake et al. [1], EFs are organized into three main components which, although interrelated, can be defined and assessed relatively independently: inhibitory control, working memory, and cognitive flexibility. Based on the initial model by Miyake et al. [1], Diamond [2] suggests different subcomponents for each EF. Thus, inhibitory control includes interference control, defined as the ability to resist distractions or irrelevant information competing with the task at hand [3], and response inhibition, understood as the ability to suppress an automatic or dominant action in favor of a behavior more appropriate to the context [1]. Working memory is associated with a verbal subcomponent, linked to the active maintenance and manipulation of linguistic information, and a non-verbal or visuospatial subcomponent, responsible for holding and manipulating spatial representations [2]. Finally, although cognitive flexibility has been linked to various subcomponents, there is consensus that switching best defines it; this refers to the ability to alternate between rules, mental frameworks, or problem-solving strategies according to task demands [1,2]. Diamond [2] further proposed that inhibitory control, working memory, and cognitive flexibility correspond to core EFs, and that from these emerge other higher-order EFs, such as reasoning, problem-solving, and planning.

EFs have been identified as relevant cognitive processes for child and adolescent development [4], and their impact has been demonstrated in different developmental areas such as academic performance, career success, and general well-being [2,5]. Several studies have described a sequential development of EFs, being more intense during childhood and slowing down in early adolescence [2,6]. However, recent longitudinal studies show that this upward trajectory is maintained throughout adolescence, with rapid progress until approximately age 15 and becoming more gradual thereafter, tending to stabilize around 18 to 20 years of age [7]. Furthermore, there is relative consensus that core EFs are the first to develop, providing the foundation for higher-order EFs, which can extend their development even into adulthood [8,9]. Importantly, when studying the development of EFs, different approaches may be taken, with an impact on both the results as well as their interpretation. Namely, direct -measuring performance at cognitive tasks- and indirect assessment -measuring behaviour through questionnaires- can be used, addressing different underlying mental constructs [10]. Moreover, studies have found different outcomes when using these two approaches [11,12].

While most individuals follow a developmental trajectory as previously described, some differ due to the presence of special educational needs (SEN). This is a broad concept that includes different developmental disorders expressed as significant difficulties in accessing learning, which require specialized support within the educational system [13].

There is ample evidence that children with SEN exhibit lower EF performance than typically developing (TD) children (e.g., [14]). Below, we present evidence on the development of EFs and their components for the most prevalent developmental difficulties of greatest interest to the present study.

### 1.1. EF and Attention Deficit Hyperactivity Disorder (ADHD)

Various investigations have evidenced EF difficulties in children with ADHD. For example, research conducted by Kofler et al. [15] in children aged 8 to 14 found that 89% of children with ADHD have difficulties in at least one EF. Of the affected EFs, the component with the greatest impact was working memory (measured with various verbal and non-verbal tasks), followed by cognitive flexibility (as measured with various switching tasks), and finally inhibitory control (measured with response inhibition tasks). Similarly, a systematic review by Sadozai et al. [16] found that children with ADHD have a moderate impairment in overall executive functioning compared to typically developing children, presenting major difficulties in the areas of attention and inhibitory control and some moderate difficulties in planning and working memory. Finally, research by Forslund et al. [17] in children aged 6 to 7 found that poor inhibitory control is associated with the presence of greater ADHD symptoms, explaining 2.8% of the sample’s variance.

### 1.2. EF and Intellectual Disability (ID)

The close relationship between intellectual functioning and EFs suggests that the impairment of the latter is inherent to intellectual disability. Therefore, a more profound intellectual limitation leads to a greater impairment of EFs [18]. A meta-analysis by Spaniol and Danielsson [19] corroborates this premise, evidencing a global, transversal, and generalized deficit in the EFs of children with ID when assessing attention, inhibitory control, and cognitive flexibility. However, recent research, such as that by Cáceres-Álvarez et al. [20], proposes that the EF profile in this group is not uniform and varies according to the degree of intellectual disability, etiology, and age. These findings suggest the existence of different profiles within the same condition. As evidence of the described variability, Sajewicz-Radtke et al. [21] identified distinct cognitive profiles in students with mild ID aged 7 to 18. The authors attribute this variability, among other influential factors, to the multifactorial origin of intellectual disability, the role played by environment and education, and comorbidity with other disorders. Despite this, verbal working memory was identified as a transversal weakness across the various age groups analyzed.

### 1.3. EF and Developmental Language Disorder (DLD)

As with the SEN already presented, there is sufficient evidence that children with DLD present difficulties in different EFs [22,23]. However, it has also been found that not all EFs are affected in the same way. For example, an empirical study in children with DLD aged 6 to 8 showed that this group presents an EF profile marked by difficulties in the interference control subcomponent, followed by problems in verbal working memory, and then difficulties in response inhibition. This study also revealed that children with DLD do not differ significantly from their typically developing peers in non-verbal working memory and cognitive flexibility (switching) [24]. A study by Blom and Boerma [25] showed that the executive deficit in DLD is mediated by age and diagnosis severity. According to this research, the interference control subcomponent is affected from age 6 due to a lack of verbal mediation, while the non-verbal working memory subcomponent is only compromised in severe profiles. Finally, a meta-analysis by Niu et al. [26] concluded that, in children with DLD, working memory deficits are mainly observed in the verbal domain and that this constitutes a strong marker of DLD, independent of age or language. Interestingly, as with other SEN, there are suggestions of individual heterogeneity in EF performance within the group of children with DLD [24,27]. Although studies adopting a person-centered approach are scarce, the existence of different executive performance profiles within the DLD group has been proposed [28].

### 1.4. EF and Learning Disabilities (LD)

Current literature suggests that children with learning disabilities (LD) present significantly lower EF performance compared to typically developing peers. For example, a study conducted by Wafa et al. [29] in children aged 6 to 13 found that the LD group presented difficulties mainly in problem-solving and planning, while the least affected component was inhibitory control. Capodieci et al. [30] concluded in their research that children diagnosed with LD show lower performance in both inhibitory control and working memory, as compared to the TD group. On the other hand, Castro et al. [31] evaluated children with dyslexia and dyscalculia (both recognized as LD), showing that both groups display reduced EF performance compared to children with TD, mainly in non-verbal working memory, inhibitory control, and cognitive flexibility (switching). However, children with dyscalculia show lower results in cognitive flexibility compared to children with dyslexia. This latest evidence highlights that heterogeneity in EF performance also exists within the LD group.

### 1.5. EF and Autism Spectrum Disorder (ASD)

Regarding Autism Spectrum Disorder (ASD), evidence indicates that EF difficulties emerge early and could be an important predictor in adaptive terms for adult life, with vulnerabilities observed from 3 years of age [32].

The literature is inconsistent regarding the reported EF profiles. For example, a study by Blijd-Hoogewys et al. [33] conducted with 127 children aged 5 to 18 found that the most affected EF is cognitive flexibility (switching), followed by inhibitory control. However, the study by Powell et al. [32] conducted on 3-year-old children diagnosed with ASD showed impairment in inhibitory control, working memory, and cognitive flexibility (switching), altering their adaptive functioning in communication, social interaction, and daily living skills. There are also no consistent results in meta-analysis studies. For instance, a meta-analysis by Cahuana et al. [34] identified an EF profile in children with ASD, marked by difficulties in working memory, cognitive flexibility, and inhibitory control (specifically in response inhibition), as well as in planning and verbal and non-verbal fluency. However, another meta-analysis conducted by Demetriou et al. [35] suggested that the deficit is global in nature, without clear distinctions between the different components and subcomponents of EFs.

In summary, although there is a wide diversity of developmental disorders grouped under the general concept of SEN, they usually coincide in that all present some EF difficulties. However, evidence regarding which EFs are most affected and which do not differ from those observed in TD children varies according to the type of SEN. Furthermore, study results are not always consistent, even when analyzing EF performance within the same type of SEN. A possible explanation for these discrepancies lies in the broad individual heterogeneity that characterizes SEN. Several authors have pointed out that individual variability in EFs within the same diagnosis can be so wide that some children with different SENs present profiles more similar to each other than to other children belonging to their own diagnostic group [16,36,37]. Based on this, a transdiagnostic perspective has been proposed, in which EFs are understood as dimensions that can be affected in various clinical profiles, rather than as specific markers of a particular disorder [16].

Consistent with this view, recent research has increasingly adopted person-centered approaches to characterize EF heterogeneity beyond traditional variable-centered comparisons. For example, Erisman and Blom [28] identified distinct latent profiles among children with DLD based on their language, phonological memory, and executive abilities, illustrating substantial heterogeneity within a single diagnostic group. Similarly, Dajani et al. [38] applied latent profile analysis (LPA) to executive measures of working memory, inhibition, and cognitive flexibility in a mixed sample of children with ASD, ADHD, comorbid ASD and ADHD, and typically developing children. They identified that the EF profiles did not reproduce diagnostic categories, providing further evidence of substantial variability in executive functioning within diagnostic groups and similarities across diagnostic boundaries. At the same time, recent meta-analytic evidence has documented substantial overlap in EF difficulties across neurodevelopmental conditions, particularly ADHD and ASD, while also identifying differences in the magnitude and pattern of difficulties across specific executive components [39,40]. Together, these findings support the value of person-centered and transdiagnostic approaches for capturing both within-diagnosis heterogeneity and cross-diagnostic similarities in executive functioning. However, person-centered and transdiagnostic research on EF has focused predominantly on specific diagnostic groups or on comparisons between a limited number of SENs, particularly ADHD and ASD. Consequently, less is known about whether common executive profiles emerge across a broader and more heterogeneous range of SEN, including conditions such as DLD, LD, and ID. Examining EF profiles across these conditions may help determine the extent to which children with different diagnoses share similar patterns of executive functioning, while also capturing heterogeneity within diagnostic categories.

Considering the above and taking the EF model by Miyake et al. [1] and Diamond’s proposal [2] as a referential framework, the present study adopts a transdiagnostic and person-centered perspective, aiming to identify EF performance profiles in a large of schoolchildren with different SENs. Given that the sample is composed of school-age children and that higher-order EFs continue to develop and differentiate during later stages [9], this study focuses exclusively on core EFs. Therefore, following the logic of previous studies analyzing EFs at the subcomponent level (e.g., [24,41]), interference control, response inhibition, verbal working memory, non-verbal working memory, and cognitive flexibility (switching) will be evaluated. Specifically, this study addresses the following research questions: (1) Is it possible to identify distinct EF performance profiles in a large sample of schoolchildren with different SENs? (2) How do these profiles relate to the different diagnoses included in the study? and (3) Is there an association between age or gender and membership in the different EF performance profiles?

## 2. Materials and Method

### 2.1. Sample

The sample consisted of 185 participants distributed across five groups (Learning difficulties, LD: *n* = 13; Attention deficit hyperactivity disorder, ADHD: *n* = 64; Intellectual disability, ID: *n* = 23; Autism Spectrum Disorder, ASD: *n* = 21; Developmental Language Disorder, DLD: *n* = 64). The mean age of the total sample was 9.8 years (SD = 2.61), with significant differences observed between groups for this variable (*p* < 0.001). The ID Group presented the highest mean age (12.65 years, SD = 1.37), while the DLD Group presented the lowest age (7.28 years, SD = 1.25). Regarding gender, 62% of the sample were boys (*n* = 115) and 38% were girls (*n* = 70). No statistically significant differences were observed in gender distribution among the groups (*p* = 0.5). See Table 1.

Participants’ diagnoses were obtained from the official records of the Special Educational Needs (SEN) support programs at their schools. In Chile, eligibility for specialized educational support requires a formal diagnostic assessment conducted by qualified professionals in accordance with regulations established by the Chilean Ministry of Education (Decree No. 170) [42]. These regulations incorporate diagnostic criteria from international classification systems, including the International Classification of Diseases (ICD) and the Diagnostic and Statistical Manual of Mental Disorders (DSM), using their most recent versions in accordance with national health and educational guidelines. Diagnostic assessments are comprehensive and interdisciplinary, and the specific procedures and professionals involved depend on the SEN diagnosis.

Specifically, ID diagnosis involves psychological, medical, and educational assessment and requires evidence of significant limitations in both intellectual functioning and adaptive behavior. Intellectual functioning is assessed using the Wechsler Intelligence Scale for Children (WISC-V), while adaptive functioning is assessed using the Vineland Adaptive Behavior Scales. ASD diagnosis involves medical assessment by a psychiatrist or neurologist, together with psychological, speech-language, and special-education assessment. ADHD diagnosis requires medical assessment, complemented by psychological and/or special-education or psychopedagogical assessment. LD diagnosis involves special-education or psychopedagogical assessment together with medical evaluation and standardized assessment of the relevant academic skills. DLD diagnosis is established through speech-language assessment as part of a multidisciplinary process involving special-education and medical professionals. Language assessment is conducted using standardized measures specified according to age by Chilean educational regulations and subsequent Ministry of Education guidelines. For school-age children from 6 years onward, language assessment includes the Instrumento de Diagnóstico para los Trastornos Específicos del Lenguaje en la Edad Escolar (IDTEL [43]).

For the purposes of the present study, participants were classified according to the primary SEN diagnosis under which they were formally admitted to the school SEN support program. Information on additional or comorbid SEN diagnoses was not systematically available in the research dataset; therefore, the presence and distribution of diagnostic comorbidities could not be examined.

Importantly, intellectual functioning is explicitly considered within this diagnostic framework. For DLD and LD, intellectual disability is considered as part of the differential diagnostic process; ADHD diagnosis also requires consideration of alternative conditions that may account for the observed difficulties. In contrast, the diagnosis of ID requires evidence of significant limitations in both intellectual functioning and adaptive behavior. According to Decree No. 170 [42], significant limitations in intellectual functioning are defined by an IQ score of 69 or below on a standardized intelligence test, and the diagnostic assessment must include information on adaptive functioning.

### 2.2. Instruments

For the evaluation of EFs, the Yellow Red battery [44] was used. Yellow Red was designed for the evaluation of inhibitory control, working memory, and cognitive flexibility. Of the seven subtests included in the battery, five were selected for the present study as indicators of these executive-function components, following the theoretical assignment reported by Rosas et al. [44]: Flies, which assesses response inhibition; Arrows, which assesses interference control; Auditory Farm, which assesses auditory—verbal—working memory; Visual Farm, which assesses visuospatial—non verbal—working memory; and Triads, which assesses cognitive flexibility.

The Yellow-Red battery was standardized in Chile using a sample of 245 children from Kindergarten to sixth grade, covering approximately 6 to 11 years of age. The standardization study explicitly examined developmental differences in performance across the age range covered by the battery, showing significant age-related increases in performance across all subtests. The battery showed adequate reliability across subtests (α ≥ 0.82), as well as significant convergent associations with established measures of executive functioning. Performance generally improved with age, although the developmental trajectories differed across tasks and some flattening of the performance curves was observed at older ages, suggesting reduced discrimination at the upper end of the age range for some subtests. This pattern was particularly evident for the Farm subtest, which showed a plateau from approximately third grade onward [44]. The validity and reliability indicators reported below for each subtest correspond to those published in the Yellow-Red standardization study [44]. In the current study, participants older than the normative data, were assigned a t-score calculated from their closest age. Namely, 11 years of age.

### 2.3. Assessments Included in the Analyses

#### 2.3.1. Inhibition

##### Interference Control

The Arrows test is a go/no-go paradigm test, in which a target arrow and 3 alternative arrows are presented, one of which must be selected according to the following rule: You must select the arrow that points to the same side as the target arrow, ignoring the color distractor. But, if the target arrow points downward, no arrow should be selected from the provided alternatives. In this way, attention is maintained, promoting inhibition when the item corresponds to the downward arrow. One point is assigned to each correct response (correct selection of arrows and inhibition of arrows pointing downward). The total number of items is 36, of which 8 correspond to inhibition items. Cronbach’s alpha for the test: 0.88. The go/no-go paradigm has been used in various studies to evaluate interference control [45,46].

##### Response Inhibition

The Flies test is a delay of gratification test. Delay of gratification tests have been related to the ability to inhibit at an early age and to predict future inhibitory capacity [47]. In this test, flying flies are presented, and the participant must squash as many as possible within a certain time. For this, a traffic light is presented, and the action must be executed only if the green light is on. If the red light is on, the participant must not squash the flies. The test lasts two minutes and has 9 red-light blocks and 9 green-light blocks, of variable length. The score is calculated by adding the number of flies squashed during the green light blocks and subtracting the sum of the flies squashed during the red-light blocks. Split-half reliability indicator: Pearson’s r = 0.82.

#### 2.3.2. Working Memory

For the evaluation of working memory, the test includes the Farm test, in its auditory-verbal and visuospatial versions. Both tests evaluate working memory in its verbal and non-verbal components, in a format similar to that applied in WISC-V [48] in the backward digit span subtest.

##### Auditory-Verbal

The Farm test in its auditory version consists of remembering the sounds of animals presented through headphones, to then select the animals to which the sounds heard correspond, selecting them in reverse order. The test begins with the sound of 2 animals and adds one animal at a time, with 2 attempts per sequence length, up to 9. Only the first sequence (of 2 animals) has four attempts. The test is suspended when the participant makes two consecutive errors at the same item length. The test is scored with one point for each correct item (sequence), with a score that can range from 0 to 20 points. A response is defined as correct only if it is given at least 200 ms after the sequence presentation.

##### Non-Verbal

The Farm test in its visual version consists of remembering the sequence of lit keys on a 3 × 3 grid, to then select the corresponding keys in reverse order. The test begins with two lit keys and adds one key at a time up to a maximum of 10. The test is suspended when the participant makes two consecutive errors at the same item length. The test is scored with one point for each correct item (sequence), with a score that can range from 0 to 18 points. A response is defined as correct only if it is given at least 200 ms after the sequence presentation. Cronbach’s alpha for the Farm test: 0.82.

#### 2.3.3. Cognitive Flexibility

Switching is evaluated through the Triads subtest, which is an adaptation of the Wisconsin Card Sorting test. In the test, four geometric figures are presented, three of which match in a characteristic: shape (square, triangle, or pentagon), color (green, yellow, blue, or red), or size (large or small). The participant must select the three that share the common characteristic. The instruction does not explicitly state the characteristics they might share, so the participant must figure it out. To support the selection of the criterion, the test provides auditory and visual feedback indicating correct and incorrect selection. The criterion changes without prior warning, and the test is suspended by category (color, shape, and size) after three consecutive errors. The test has 21 items, 5 of which correspond to the color criterion, 5 to the shape criterion, and 5 to the size criterion. Finally, the test ends with a mixed phase, meaning it can present items from any of the three categories. Participants have 3 chances to answer each item. If they fail all three attempts, the category is suspended. One point is awarded for each correct answer on the first attempt, and then points are deducted in successive attempts (per item). If correctly answered on the second attempt, they receive 0.6 points, and 0.3 points on the third attempt. Cronbach’s alpha: 0.86.

### 2.4. Procedure

Assessments were conducted during school hours, with prior approval from the educational institution, informed consent from parents/guardians, and informed assent from participants. The EF assessment was carried out in a session of approximately 40 min. Participants also completed additional assessments as part of the broader research protocol, which are not reported in the present study. The study was conducted with the approval of the University Institutional Review Board (IRB).

Participants were selected from a general sample in educational establishments in the Metropolitan and Maule Regions in Chile. The diagnosis was provided by the SEN support team at the educational establishments they attend. In Chile, for students to access specialist support to address SEN, they must be diagnosed using a battery of tests and evaluations by specialists (MINEDUC, Decree No. 170 [42]). Furthermore, depending on the characteristics of the SEN, the diagnostic team may include special educators, speech therapists, neurologists, among others.

### 2.5. Statistical Analyses

Initially, descriptive analyses were performed for each variable (interference control, response inhibition, verbal and non-verbal working memory, cognitive flexibility) in the total sample, as well as in each group according to SEN diagnosis. Accuracy (raw score) on the Yellow Red battery tasks [44] was used as a measure for each EF. Considering that all variables (EF subcomponents) are numerical, a mean and standard deviation were calculated, calculating the difference between groups (diagnosis and gender) using an ANOVA.

Next, to explore the correlation between the different EFs, a Spearman test was used. This was done to avoid including highly correlated tests, which is a difficulty when performing LPA.

To answer the first research question, related to the identification of EF profiles in children with SEN, an LPA was carried out on the complete sample without separating diagnoses (*n* = 185). This is an exploratory technique that allows individuals to be classified into unobserved subgroups based on response patterns in a specific set of observed variables [49]. The LPA was exploratory and data-driven, with the number of profiles determined based on model-fit indices and the theoretical interpretability of the resulting profiles.

For the LPA analyses, each raw score of EF performance was transformed into a T-score. This type of score corresponds to a standardized measure with a mean of 50 and a standard deviation of 10 and was calculated from the normative data of the Yellow Red instrument [44]. The use of T-scores allowed standardizing the results of tests that originally present different scales, ensuring that all measures would be expressed in the same metric. Likewise, this methodological decision reduced the influence of age-related differences in raw performance by expressing participants’ EF performance relative to the corresponding normative reference group Finally, the use of T-scores facilitates the clinical interpretation of the results, since the participants’ performance is interpreted in relation to the general reference population, without SEN.

Before running the LPA analysis, univariate and multivariate outlier analyses were performed. Namely, univariate outlier screening and Mahalanobis distance (D^2^).

A series of LPA was specified using a step-by-step procedure, starting with a one-profile model and progressively increasing to a 5-profile model. This analysis was performed by defining profiles with equal variances (model 1) and varying variances (model 2). The five EFs (interference control-cognitive inhibition, CI-, response inhibition -BI-, verbal WM-VWM-, non-verbal WM-NVWM-, and cognitive flexibility) were used as indicators for the profiles. To determine the model solution that best fit the data, a combination of model fit indices was used. Fit statistics included the Akaike Information Criterion (AIC) and the Bayesian Information Criterion (BIC), and Sample-Size Adjusted Bayesian Information Criterion (SABIC). Lower AIC, BIC and SABIC values indicate a better model fit [49]. Entropy was also calculated, a metric that evaluates the degree to which individuals are correctly classified into the latent classes. Higher entropy values represent greater certainty in classification, and values close to 0.80 indicate that the classes are discriminant [47]. Additionally, the minimum average probability with which the model correctly predicts membership in each class was calculated. Values above 0.80 are considered optimal [47]. The solution with the number of classes that showed the best fit according to all the above indicators was graphed and interpreted from a theoretical standpoint.

To answer the second research question—about the relationship between the emerging profiles from the objective 1 analysis and the diagnoses defined by professionals—the difference in the number of participants per diagnosis assigned to each profile was analyzed. To examine this, Fisher’s Exact test was conducted to determine if there is an association between the profiles obtained by the LPA and the diagnoses determined by the professionals.

Finally, to respond to objective 3—about the relationship between membership in the emerging profiles and age and gender—an analysis of variance (ANOVA) was performed.

Analyses were conducted using Rstudio software (version 2026.01.0+392; R version 4.5.2), and the packages: tidyLPA, dplyr, ggplot2. The study and analysis plan were not preregistered.

## 3. Results

### 3.1. Descriptive Analyses

Before group comparisons, distribution normality and homogeneity of variance were evaluated using Shapiro-Wilk and Levene’s tests, respectively. As the majority of subtests violated the assumption of homoscedasticity (Levene’s *p* < 0.05), Welch’s ANOVA was conducted to examine group differences, followed by Games-Howell post-hoc tests for pairwise comparisons (see Appendix A). Table 2 shows that groups differ in performance, at all subtests. Pairwise comparisons showed that all groups significantly differ. Visual inspection of the means shows that the ID group exhibits the lowest performance across the evaluated EFs. In contrast, the ASD group shows mean scores—yet statistically significant—closer to those of the ADHD and LD. Additionally, participants presenting LD, ADHD, and DLD show higher results among the studied groups in all EFs, with statistically significant differences among them as well. Furthermore, in general, a high data dispersion can be observed, reflected in elevated standard deviations. Sensitivity analyses excluding participants over the age of 11 are shown in Appendix B.

Regarding gender, the results show no differences between boys and girls in any of the subtests.

### 3.2. EF Profiles

The latent profile analysis was performed by comparing zero covariance models (model 1) and variable variance models (model 2). For each type of model, models considering 1 to 5 latent profiles were calculated. The best model was selected based on lower AIC, BIC and SABIC, as well as higher Entropy (over a 0.7 threshold). A summary of the indicators for the models can be found in Appendix C. Importantly, no outliers were detected with at least one subtest with |Z| > 3.29, or D2 ≥ 20.51 (df = 5, *p* < 0.001).

The optimal model proved to be model 2 (variable covariance) and 4 latent profiles, described as: Specific inhibition deficit (SID, Profile 1), Severe executive deficit (SED, Profile 2), Mild executive deficit (MED, Profile 3), and Average executive performance (AEP, Profile 4). Figure 1 shows the latent profiles emerging from the selected model. Furthermore, Table 3 presents means and standard deviations of the results for participants assigned to each profile. Sensitivity analysis, excluding participants in the ID group, is presented in Appendix D.

### 3.3. Profile Composition

Table 4 and Table 5 show the distribution of diagnoses across the different identified profiles. It is observed that the SID profile is composed of 9.09% participants diagnosed with LD, 40.91% diagnosed with ADHD, only 3.03% participants diagnosed with ID, 9.09% diagnosed with DLD, and 37.88% diagnosed with ASD. Meanwhile, for the SED profile, 70% correspond to participants diagnosed with ID, 5% to participants diagnosed with LD, 5% diagnosed with ADHD, 10% diagnosed with DLD, and 10% diagnosed with ASD. In the case of the MED profile, 4% corresponds to participants diagnosed with LD, 20% to participants diagnosed with ADHD, 20% diagnosed with ID, 16% diagnosed with DLD, and 40% diagnosed with ASD. Finally, the AEP profile is composed of 6.76% participants with LD, 41.89% participants with ADHD, 2.7% participants diagnosed with ID, 12.16% participants diagnosed with DLD, and 36.49% participants with ASD. The Fisher’s exact test, with Monte Carlo simulation (10,000 replicates) revealed a significant association between diagnosis and membership in a particular profile (*p* < 0.001), suggesting a tendency for some diagnoses to concentrate in specific profiles.

When running Welch’s ANOVA, the obtained profiles show significant differences based on participants’ age (F = 16.916, df = 3, *p* < 0.001, η^2^ = 0.134). Post hoc analyses showed that the difference is significant between the profile SED, and all the other profiles (SID, MED and AEP) (Figure 2). On the other hand, the analysis of the relationship between latent profiles and gender showed no differences. The result of the χ^2^ test showed no significant relationship between gender and the profile to which the participant is assigned (χ^2^ = 2.68, df = 3, *p* = 0.44).

## 4. Discussion

The present study adopted a transdiagnostic and person-centered perspective to explore the existence of emerging profiles from the assessment of core EFs in schoolchildren with various Special Educational Needs (SEN): Learning Difficulties (LD), Attention Deficit Hyperactivity Disorder (ADHD), Intellectual Disability (ID), Autism Spectrum Disorder (ASD), and Developmental Language Disorder (DLD). Through Latent Profile Analysis (LPA) modeling, and based on the EF model by Miyake et al. [1] and Diamond’s [2] specifications by subcomponents, interference control, response inhibition, auditory-verbal and non-verbal working memory, and cognitive flexibility were evaluated using age-standardized T-scores.

The first research question aimed to determine whether it was possible to identify distinct EF profiles in a large and heterogeneous sample of schoolchildren with different SENs. The results support this idea of distinct executive performance profiles across the entire sample, as the latent profile analysis identified four profiles: Specific Inhibition Deficit (SID; Profile 1, *n* = 66), Severe Executive Deficit (SED; Profile 2, *n* = 20), Mild Executive Deficit (MED; Profile 3, *n* = 25), and Average Executive Performance (AEP; Profile 4, *n* = 74). Considering that 4 executive profiles emerged from the data and that 5 SENs were considered in the sample, these results support person-centered approaches, proposing that cognitive differences can be organized into performance patterns that do not necessarily align with traditional diagnostic categories [16,50]. Due to the characteristics of the study, an important factor to consider is that the SEN groups are heterogeneous in their sizes. For instance, the one with a diagnosis of Learning Disabilities has the smallest sample size (*n* = 13), so its distribution across the different latent profiles should be interpreted with caution. Furthermore, it is suggested to consider these 4 profiles as descriptive classifications rather than diagnostic labels.

A particularly relevant aspect is that the identified profiles did not differ solely in severity. While the Severe Executive Deficit (Profile 2) and Mild Executive Deficit (Profile 3) profiles seem to reflect different degrees of executive impairment, the Specific Inhibition Deficit profile (Profile 1) showed a qualitatively different pattern, characterized by specific difficulties in the cognitive inhibition subcomponent, with relatively preserved performance in the other executive components. However, these differences should be interpreted considering the methodological limitation of using single indicators for each profile. Namely, as each subcomponent is represented by a single task, task impurity could influence the distinction of these profiles. For this reason, the characterization of specific latent profiles, such as that of Specific Inhibition Deficit demands caution. Furthermore, the said limitation poses the need for future research to use multiple indicators per domain to isolate the specific variance of each executive function more rigorously.

Another relevant reflection emerging from these findings is the importance of considering EF assessment at the subcomponent level to accurately describe cognitive profiles in children with SEN. Measuring distinct subcomponents allows identifying profiles with specific deficits that would otherwise remain hidden under a global index of executive functioning. This supports the notion that EFs constitute a set of partially differentiated skills rather than a single unitary construct [1,2]. Furthermore, it can be relevant in children with SEN considering that they have been described as having variable profiles across different sub-skills [15,24]. Thus, the evaluated schoolchildren do not present a generalized deficit but show specific difficulties in certain aspects. For example, in the case of the Specific Inhibition Deficit profile, they present a difficulty in inhibitory control, but only in the interference control subcomponent, while maintaining adequate and relatively homogeneous performance in other areas, such as working memory and cognitive flexibility and, interestingly, in the response inhibition subcomponent.

The second research question addressed the relationship between the identified EF profiles and the clinical diagnoses present in the sample. The results showed that there is a relationship between diagnoses and emergent profiles, since most executive profiles mainly concentrate in two diagnostic categories. This is most evident for the Severe Executive Deficit profile, as 70% of it is made up of children with intellectual disability. These findings are consistent with the literature documenting generalized EF difficulties in this population [18,19]. Along with the above, the obtained results also show that while the relationship between profiles and diagnoses exists, it is not perfect. The fact that the same executive profile concentrates at least two diagnostic categories means that some children presenting different SENs demonstrate similar executive performance. In line with the discussion raised in the first part, these results highlight the complementary value of a transdiagnostic perspective for characterizing executive functioning beyond diagnostic categories alone. Moreover, observing how children with a given diagnosis are distributed within the executive profiles reveals that there is heterogeneity within each SEN. In the case of DLD, this seems to be particularly marked. The results indicate that all children with DLD are mainly distributed across three executive profiles: Specific Inhibition Deficit, Mild Executive Deficit, and Average, Executive Performance. In line with these findings, several studies have suggested that high variability in executive performance is a characteristic of children with DLD [24,27].

One executive profile worth analyzing with particular emphasis, the Average Executive Performance profile. As its name indicates, it represents children whose standardized scores were within the average range across EF subcomponents relative to the Yellow-Red normative reference sample. Therefore, the term “average” should not be interpreted as evidence of equivalent performance to a concurrently assessed TD control group. It is striking that this profile is composed of a high proportion of children with ADHD and ASD (41.89% and 36.49% respectively). These findings are consistent with previous studies indicating that within these diagnostic groups, there are also children with relatively preserved executive performance [15,35].

In summary, the findings addressing the second research question show that diagnoses can share common executive mechanisms and, at the same time, display considerable internal variability.

The third research question sought to examine whether demographic variables such as age and gender are associated with membership in the different identified profiles. The results indicated that, for age, there is a significant difference between SED profile and the rest. On the other hand, no difference was found between gender and profile. Regarding gender, the absence of differences is consistent with part of the literature that has shown small or inconsistent effects on EFs between males and females [7]. Although some studies report slight advantages for girls in self-regulation or inhibitory control tasks due to differences in maturational rates, these effects tend to dilute with age and are usually not robust or consistent [16].

As for age, the difference found is most probably driven by the high proportion of ID participants in SED profile. In is worth noting, that the ID group is the older group, with a mean age of 12.65 years, probably pushing this significant difference reported. Thus, in this sample of schoolchildren with SEN, chronological age was not significantly associated with three out of four profile membership. In other words, children of similar ages presented different executive profiles, while participants of different ages could share comparable standardized EF performance patterns, with the sole exception of the SED profile. This finding should be interpreted considering that the profiles were derived from age-standardized scores, which reduce age-related differences in raw performance by expressing participants’ performance relative to normative expectations. However, standardization does not necessarily eliminate all developmental differences in the measurement or interpretation of EF performance. Therefore, the absence of significant age differences across profiles should not be interpreted as evidence that age does not influence executive functioning, but rather that chronological age was not significantly associated with profile membership in this particular sample.

Overall, the results support the relevance of a transdiagnostic perspective on EFs, while also showing that diagnostic categories remain meaningfully associated with executive-profile membership. Thus, categorical diagnoses and dimensional EF profiles appear to provide partially overlapping but non-equivalent information. Whereas diagnostic categories were significantly associated with profile membership, the variability observed within diagnoses and the presence of similar EF profiles across different diagnostic groups suggest that executive profiling may provide complementary information by capturing individual differences that are not fully represented by diagnostic categories alone. These observations may have relevant implications for research and educational practice, suggesting that individual executive profiles could be considered alongside diagnostic information when identifying students’ specific support needs. However, the present findings do not establish that interventions based on executive profiles are more effective than those informed by diagnostic categories.

The findings presented here must be interpreted considering several limitations. First, individual measures of intellectual and adaptive functioning obtained during the diagnostic process, as well as information regarding ID severity, were not available in the present dataset. Therefore, although these variables are considered within the formal diagnostic procedures described above, we were unable to examine their association with EF performance or include general intellectual functioning as a covariate. Although intellectual functioning is considered within the formal diagnostic process, the absence of individual cognitive scores prevents us from determining whether, and to what extent, differences in general cognitive functioning may have contributed to the observed EF profiles. d A further limitation concerns the lack of systematic information on diagnostic comorbidity. Participants were classified according to the primary SEN diagnosis under which they were formally admitted to the school SEN support program; however, information on additional SEN diagnoses was not available in the research dataset. Therefore, we cannot rule out that comorbid conditions may have partly contributed to the overlap observed across executive profiles. Another limitation concerns the substantial age differences across diagnostic groups. Although the use of standardized T-scores reduced the influence of age-related differences in raw EF performance, standardization cannot be assumed to eliminate all developmental differences in the measurement properties or discriminative capacity of the tasks. Other limitations include unequal group sizes, the cross-sectional nature of the design, and the exclusive use of direct assessment measures. We highlight here that a previous study reported differences in the results obtained through direct and indirect tests in participants with ASD and ADHD [12]. Another limitation is the absence of a concurrently assessed TD control group. Consequently, comparisons with typical performance relied on the external normative reference sample of the Yellow-Red battery rather than on a control group assessed under the same study conditions. This should be considered particularly when interpreting the Average Executive Performance profile, which reflects performance within the average normative range rather than demonstrated equivalence to a concurrently assessed TD group. In addition, future studies should incorporate multiple measures of each EF component to strengthen the assessment of the executive domains examined and reduce the potential influence of task-specific characteristics. Finally, future research should incorporate longitudinal designs, consider contextual variables, and examine the relationship between executive profiles and functional outcomes to advance toward a more ecological and applied understanding of executive functioning. At this point, new questions also arise to distinguish between groups that were not classified when using only direct assessment instruments. For future studies, indirect assessment instruments could be incorporated into the assessment battery to help describe the executive functioning of participants with diagnoses of high educational interest, such as ADHD and ASD.

## 5. Conclusions

The present study shows that, by using a trans-diagnostic, person-centred approach, executive function in children with SEN configures heterogeneous cognitive profiles that transcend diagnostic labels. These findings highlight the importance of prioritising individual cognitive profiles over traditional clinical categories, when creating and implementing educational interventions. Nonetheless, these findings should be interpreted in light of the study’s limitations, such as the lack of indirect and intellectual functioning assessments and the absence of a typically developing group as a control group. Therefore, both the findings and limitations invite for further research into the transdiagnostic person-centred approach, with additional assessments, broader and more diverse samples and longitudinal data.

## Figures and Tables

**Figure 1:**
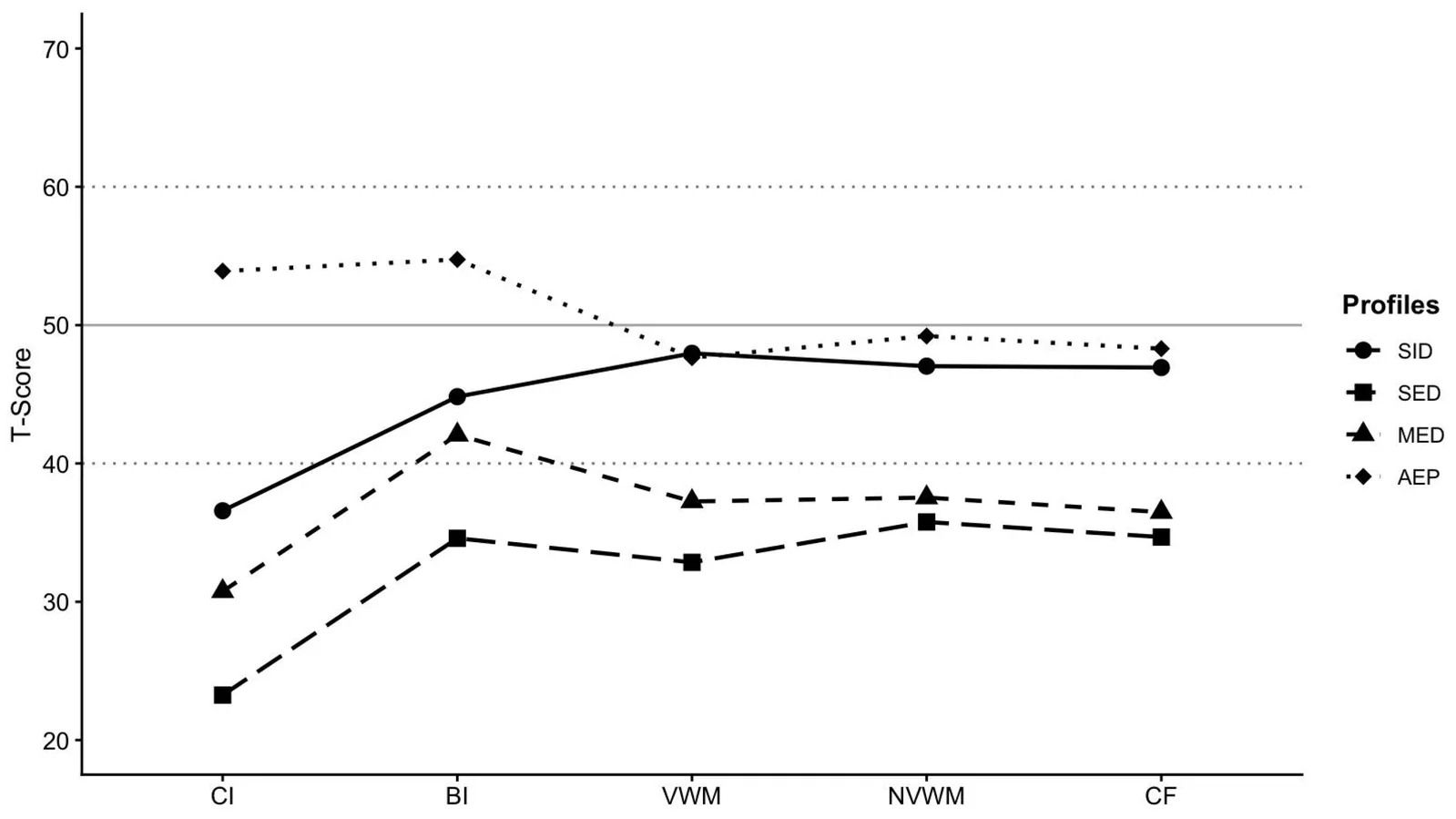
Emergent Latent Profiles. T-scores results at Yellow Red are shown by subtest. Note: CI: Cognitive inhibition; BI: Behavioural Inhibition; VWM: Verbal Working Memory; NVWM: Non Verbal Working Memory; CF: Cognitive Flexibility; SID: Specific inhibition deficit; SED: Severe executive deficit; MED: Mild executive deficit; AEP: Average executive performance. T-score obtained from standardization sample.

**Figure 2:**
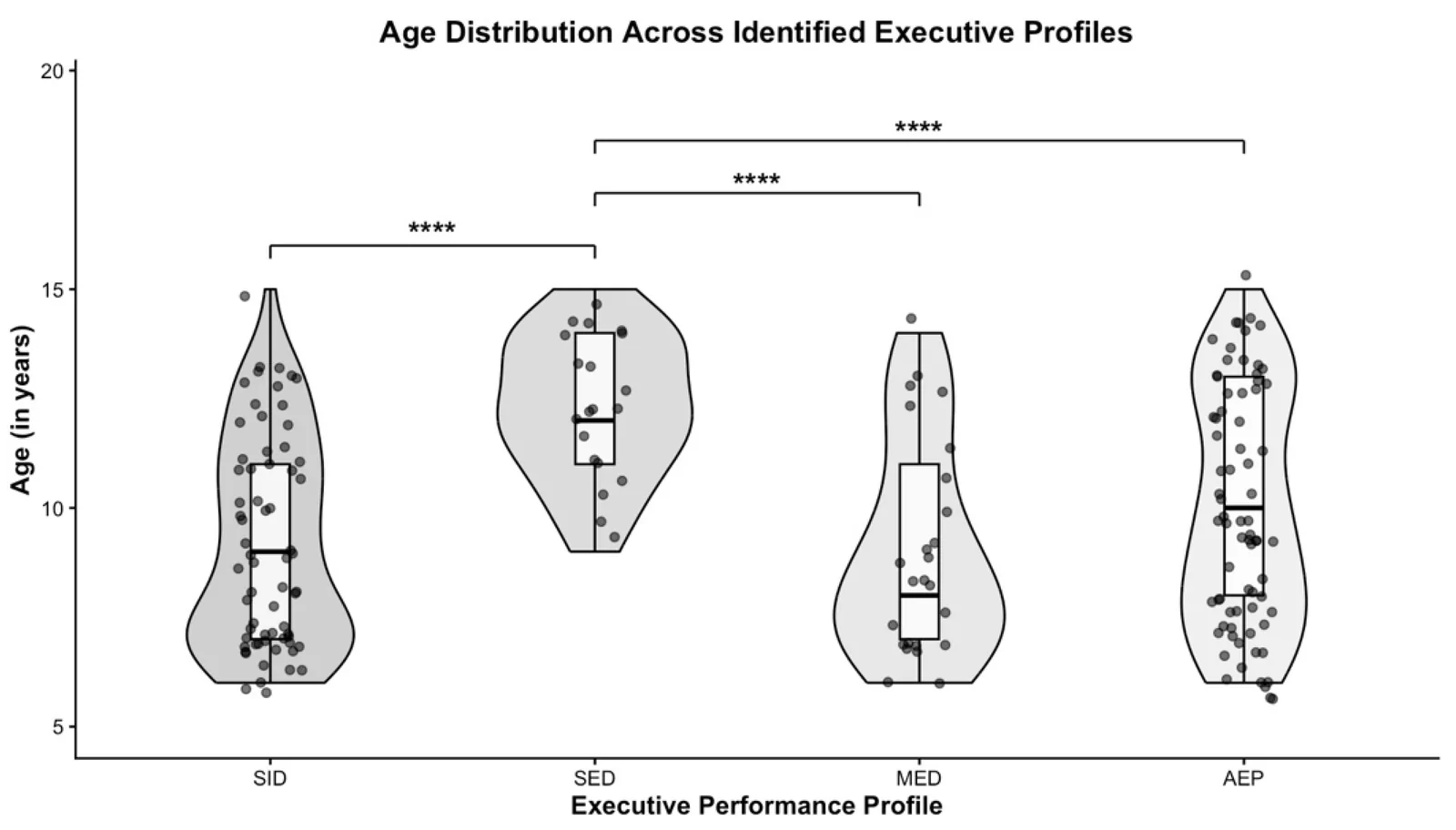
Welch’s ANOVA analysis of age difference between emergent profiles. Note: SID: Specific inhibition deficit; SED: Severe executive deficit; MED: Mild executive deficit; AEP: Average executive performance. **** *p* < 0.001 Pairwise comparison using Games-Howell test.

**Table 1:** Sociodemographic characterization of the sample by diagnostic group.

	Sample (N = 185)	ADHD (N = 64)	ID (N = 23)	DLD (N = 64)	LD (N = 13)	ASD (N = 21)	*p*-Value
Age in years ± (SD)	9.80 (2.61)	10.92 (2.16)	12.65 (1.37)	7.28 (1.25)	10.38 (2.06)	10.57 (1.96)	<0.001 ^1^
Gender, *n* (%)							0.5 ^2^
Female	70 (38%)	25 (39%)	8 (35%)	25 (39%)	7 (54%)	5 (24%)	
Male	115 (62%)	39 (61%)	15 (65%)	39 (61%)	6 (46%)	16 (76%)	

^1^ *p*-value from one-way ANOVA. ^2^ *p*-value from Fisher’s exact test. Note: ADHD: Attention deficit hyperactivity disorder; ID: Intellectual Disability; DLD: Developmental Language Disorder; LD: Learning disabilities; ASD: Autism Spectrum Disorder.

**Table 2:** Mean (and standard deviation) standardized results at Yellow Red subtests, by SEN type. A group comparison with Welch’s ANOVA is shown for diagnostic, and Welch’s independent samples *t*-test, for gender group comparisons.

Subtest	Range Raw	Range Std	ADHD	ID	DLD	LD	ASD	F	η^2^	q Value	Female	Male	t Value	q Value
			*n* = 64	*n* = 23	*n* = 64	*n* = 13	*n* = 21				*n* = 70	*n* = 115		
CI	7–36	22–66	43.72 (12.11)	27.75 (9.01)	42.65 (11.50)	44.84 (11.97)	42.30 (13.42)	13.14	0.166	<0.001	40.91 (12.92)	41.51 (12.58)	−0.31	0.950
BI	0–160	20–71	49.64 (9.52)	35.40 (11.18)	48.55 (9.16)	46.85 (10.97)	49.79 (12.78)	7.6	0.172	<0.001	45.86 (10.65)	48.20 (11.18)	−1.42	0.392
VWM	0–11	32–72	46.71 (8.61)	34.31 (3.24)	45.95 (9.44)	46.95 (8.48)	45.24 (8.00)	36.81	0.187	<0.001	43.37 (7.78)	45.60 (9.90)	−1.7	0.392
NVWM	0–12	32–75	47.86 (9.63)	37.44 (5.66)	44.75 (8.45)	49.48 (11.77)	46.08 (8.27)	11.03	0.13	<0.001	45.31 (9.17)	45.46 (9.54)	−0.1	0.950
CF	0–21	20–72	46.90 (9.97)	35.17 (7.91)	46.80 (10.56)	47.82 (7.72)	40.46 (11.02)	10.56	0.152	<0.001	44.68 (10.55)	44.78 (10.81)	−006	0.950

Mean (SD). One-way analyses of means. Note: Total sample = 185. q-values show False Discovery Rate (FDR)-adjusted *p*-values by Benjamini-Hochberg procedure. ADHD: Attention deficit hyperactivity disorder; ID: Intellectual Disability; DLD: Developmental Language Disorder; LD: Learning disabilities; ASD: Autism Spectrum Disorder. CI: Cognitive inhibition-Arrows; BI: Behavioural Inhibition-Flies; VWM: Verbal Working Memory-Auditory Farm; NVWM: Non Verbal Working Memory-Visual Farm; CF: Cognitive Flexibility-Triads.

**Table 3:** Mean performance, and standard deviation, at Yellow red subtest by Latent Profile.

LP		CI		BI		VWM		NVWM		CF	
	*n*	$\bar{x}$	SD	$\bar{x}$	SD	$\bar{x}$	SD	$\bar{x}$	SD	$\bar{x}$	SD
SID	66	36.57	7.97	44.83	8.41	47.96	7.67	47.03	9.34	46.93	9.9
SED	20	23.26	1.24	34.59	10.61	32.85	0.22	35.77	3.09	34.68	9.15
MED	25	30.75	5.26	42.07	11.52	37.25	3.17	37.53	3.59	36.48	8.52
AEP	74	53.90	5.54	54.74	7.37	47.65	9.04	49.21	8.57	48.29	9.29

Note: CI: Cognitive inhibition-Arrows; BI: Behavioural Inhibition-Flies; VWM: Verbal Working Memory-Auditory Farm; NVWM: Non Verbal Working Memory-Visual Farm; CF: Cognitive Flexibility-Triads; SID: Specific inhibition deficit; SED: Severe executive deficit; MED: Mild executive deficit; AEP: Average executive performance.

**Table 4:** Number of participants (and percentage) per diagnosis within each identified profile.

Profile	ADHD	ID	DLD	LD	ASD
SID	27 (40.91%)	2 (3.03%)	25 (37.88%)	6 (9.09%)	6 (9.09%)
SED	1 (5%)	14 (70%)	2 (10%)	1 (5%)	2 (10%)
MED	5 (20%)	5 (20%)	10 (40%)	1 (4%)	4 (16%)
AEP	31 (41.89%)	2 (2.7%)	27 (36.49%)	5 (6.76%)	9 (12.16%)

Note: SID: Specific inhibition deficit; SED: Severe executive deficit; MED: Mild executive deficit; AEP: Average executive performance.

**Table 5:** Number of participants (and percentage) assigned to each profile per diagnosis.

Profile	SID	SED	MED	AEP
ADHD	27 (42.19%)	1 (1.56%)	5 (7.81%)	31 (48.44%)
ID	2 (8.7%)	14 (60.87%)	5 (21.74%)	2 (8.7%)
DLD	25 (39.06%)	2 (3.12%)	10 (15.62%)	27 (42.19%)
LD	6 (46.15%)	1 (7.69%)	1 (7.69%)	5 (38.46%)
ASD	6 (28.57%)	2 (9.52%)	4 (19.05%)	9 (42.86%)

Note: SID: Specific inhibition deficit; SED: Severe executive deficit; MED: Mild executive deficit; AEP: Average executive performance.

**Figure A1:**
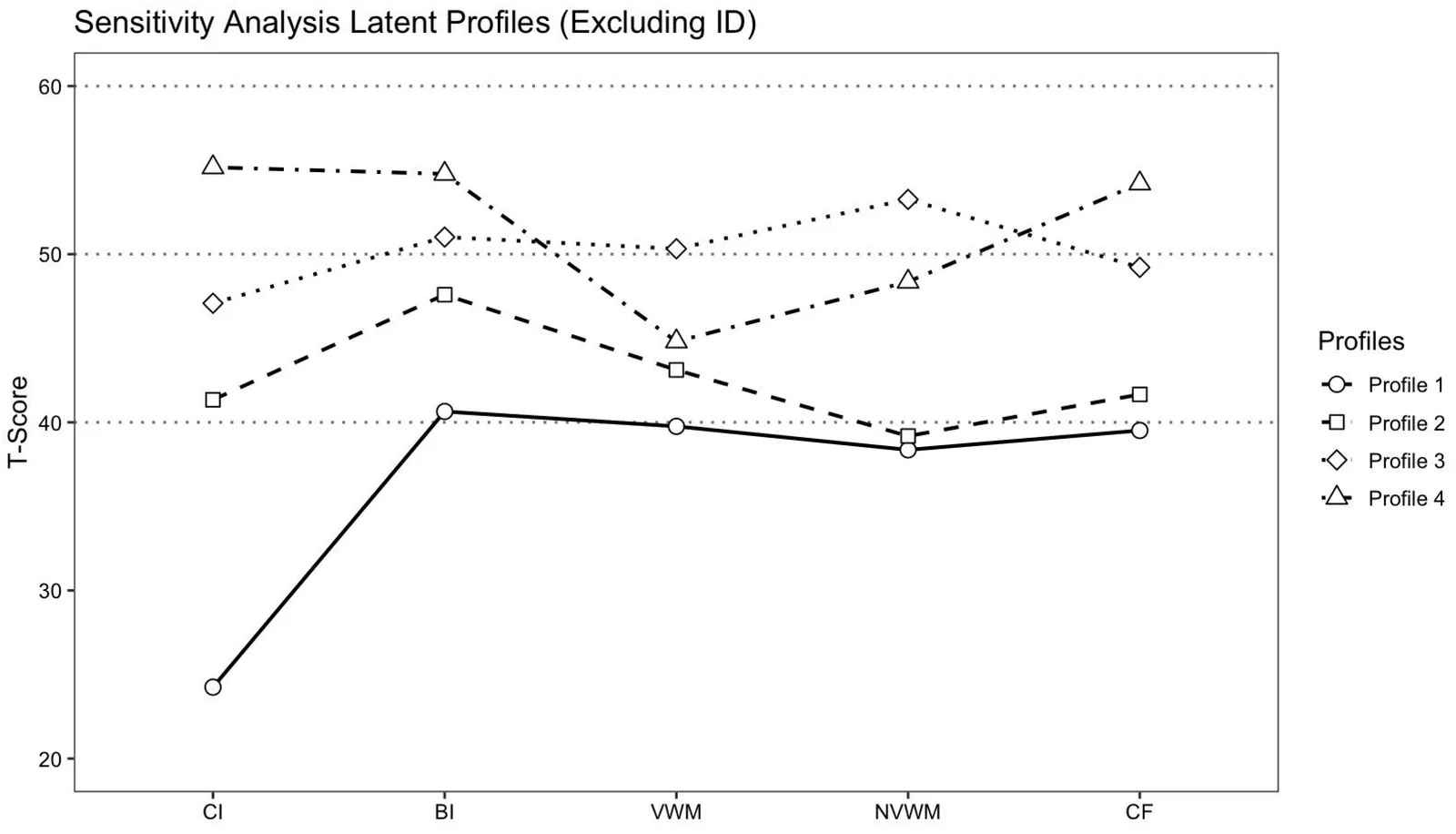
Panel 3: Emergent profiles excluding students with ID.

## Data Availability

The data presented in this study are available on request from the corresponding author due to confidentiality agreements with the participants.

## Abbreviations

The following abbreviations are used in this manuscript:

ADHD	Attention deficit hyperactivity disorder
ID	Intellectual Disability
DLD	Developmental Language Disorder
LD	Learning disabilities
ASD	Autism Spectrum Disorder
CI	Cognitive inhibition
BI	Behavioural Inhibition
VWM	Verbal Working Memory
NVWM	Non Verbal Working Memory
CF	Cognitive Flexibility
SID	Specific inhibition deficit
SED	Severe executive deficit
MED	Mild executive deficit
AEP	Average executive performance

## Appendix A

**Table A1:** Games-Howell’s pairwise analyses.

Prueba	group1	group2	estimate	p.adj	p.adj.signif
CI std	ADHD	ID	−15.97063179	0.000000197	****
CI std	ID	DLD	14.90485054	0.00000077	****
CI std	ID	LD	17.09622074	0.002	**
CI std	ID	ASD	14.55391304	0.002	**
VWM std	ADHD	ID	−12.40012908	0.000000000195	****
VWM std	ID	DLD	11.64591033	0.00000000021	****
VWM std	ID	LD	12.64280936	0.001	***
VWM std	ID	ASD	10.93863354	0.0000343	****
NVWM std	ADHD	ID	−10.42712636	0.00000043	****
NVWM std	ID	DLD	7.319626359	0.000204	***
NVWM std	ID	LD	12.04401338	0.024	*
NVWM std	ID	ASD	8.647163561	0.003	**
BI std	ADHD	ID	−14.24164402	0.0000428	****
BI std	ID	DLD	13.14461277	0.000141	***
BI std	ID	LD	11.44772575	0.045	*
BI std	ID	ASD	14.38457557	0.003	**
CF std	ADHD	ID	−11.73165082	0.00000727	****
CF std	ID	DLD	11.63071332	0.0000112	****
CF std	ID	LD	12.65471572	0.000711	***

Note: * *p* < 0.05, ** *p* < 0.01, *** *p* < 0.001, **** *p* < 0.0001.

## Appendix B

**Table A2:** Sensitivity analyses of sample. Welch’s ANOVA and Fisher’s exact test, excluding participants over the age of 11.

Characteristic	Overall (N = 128)	ADHD (N = 37)	ID (N = 5)	DLD (N = 63)	LD (N = 9)	ASD (N = 14)	*p*-Value
**Age in years**							<0.001 ^1^
6	15 (12%)	0 (0%)	0 (0%)	15 (24%)	0 (0%)	0 (0%)	
7	36 (28%)	4 (11%)	0 (0%)	32 (51%)	0 (0%)	0 (0%)	
8	21 (16%)	7 (19%)	0 (0%)	9 (14%)	3 (33%)	2 (14%)	
9	20 (16%)	7 (19%)	0 (0%)	3 (5%)	3 (33%)	7 (50%)	
10	17 (13%)	9 (24%)	2 (40%)	3 (5%)	1 (11%)	2 (14%)	
11	19 (15%)	10 (27%)	3 (60%)	1 (2%)	2 (22%)	3 (21%)	
**Gender**							0.2 ^2^
Female	46 (36%)	11 (30%)	1 (20%)	25 (40%)	6 (67%)	3 (21%)	
Male	82 (64%)	26 (70%)	4 (80%)	38 (60%)	3 (33%)	11 (79%)	

Note: ^1^ Welch’s ANOVA *p*-Value; ^2^ Fischer’s exact test *p*-Value.

## Appendix C

**Table A3:** Latent Profile Analysis Model Fit and Classification Indices for Cognitive Indicators.

Model	Classes	LogLik	AIC	AWE	BIC	SABIC	CAIC	CLC	KIC	ICL	Entropy	prob_min	prob_max	*n*_min	*n*_max
1	1	−3486.480705	6992.96141	7105.368527	7025.164969	6993.491958	7035.164969	6974.96141	7005.96141	−7025.164969	1	1	1	1	1
1	2	−3400.639431	6833.278861	7014.721982	6884.804554	6834.127738	6900.804554	6802.887127	6852.278861	−6908.821659	0.8041328501	0.9280401535	0.9536025092	0.3513513514	0.6486486486
1	3	−3384.558175	6813.11635	7063.270635	6883.964178	6814.283555	6905.964178	6770.657721	6838.11635	−6930.452756	0.7706854835	0.8082814183	0.9429059255	0.2594594595	0.4594594595
1	4	−3375.833798	6807.667596	7126.498977	6897.837559	6809.15313	6925.837559	6753.176142	6838.667596	−6962.515185	0.754272908	0.6893506169	0.9297324611	0.0972972973	0.4594594595
1	5	−3364.973429	6797.946858	7185.465228	6907.438956	6799.750721	6941.438956	6731.412684	6834.946858	−6987.411587	0.7329129802	0.7422227889	0.89418643	0.0972972973	0.3297297297
2	1	−3486.480705	6992.96141	7105.368527	7025.164969	6993.491958	7035.164969	6974.96141	7005.96141	−7025.164969	1	1	1	1	1
2	2	−3357.791776	6757.583553	6995.91766	6825.211025	6758.697703	6846.211025	6717.50439	6781.583553	−6828.386092	0.9604187661	0.9823946482	0.9941395117	0.1891891892	0.8108108108
2	3	−3337.119045	6738.23809	7102.781185	6841.289476	6739.935843	6873.289476	6675.797767	6773.23809	−6883.084199	0.7798387388	0.8471070766	0.9971223388	0.1891891892	0.4108108108
**2**	**4**	**−3289.114056**	**6664.228113**	**7154.613381**	**6802.703413**	**6666.509469**	**6845.703413**	**6579.793446**	**6710.228113**	**−6857.197428**	**0.7826663995**	**0.8072315721**	**0.9981349812**	**0.1081081081**	**0.4**
2	5	−3276.372138	6660.744277	7276.99432	6834.643491	6663.609235	6888.643491	6554.292663	6717.744277	−6902.651967	0.7741929744	0.766323979	0.9976480973	0.1081081081	0.3405405405

Note: The selected model is presented in bold.

## Appendix D

**Table A4:** Panel 1 Sensitivity analysis of Latent Profile Analysis, excluding participants in the ID group.

Model	Classes	LogLik	AIC	AWE	BIC	CAIC	CLC	KIC	SABIC	ICL	Entropy	prob_min	prob_max	*n*_min	*n*_max
1	1	−3011.64	6043.274	6153.026	6074.15	6084.15	6025.274	6056.274	6042.492	−6074.15	1	1	1	1	1
1	2	−2970.84	5973.679	6151.061	6023.08	6039.08	5943.1	5992.679	5972.428	−6055.77	0.710537	0.840136	0.953736	0.314815	0.685185
1	3	−2959.51	5963.016	6207.59	6030.943	6052.943	5920.295	5988.016	5961.295	−6094.62	0.639953	0.753586	0.918	0.296296	0.351852
1	4	−2950.75	5957.491	6269.007	6043.944	6071.944	5902.88	5988.491	5955.302	−6109.05	0.694465	0.769266	0.908318	0.12963	0.339506
1	5	−2945.83	5959.652	6338.206	6064.63	6098.63	5893.055	5996.652	5956.994	−6140.64	0.701298	0.74134	0.924871	0.098765	0.246914
2	1	−3011.64	6043.274	6153.026	6074.15	6084.15	6025.274	6056.274	6042.492	−6074.15	1	1	1	1	1
2	2	−2954.56	5951.128	6184.104	6015.968	6036.968	5910.832	5975.128	5949.487	−6031.08	0.851543	0.916667	0.972874	0.240741	0.759259
2	3	−2943.63	5951.259	6307.515	6050.062	6082.062	5888.609	5986.259	5948.757	−6109.01	0.675065	0.801876	0.913537	0.253086	0.444444
**2**	**4**	**−2912.21**	**5910.413**	**6389.331**	**6043.18**	**6086.18**	**5826.029**	**5956.413**	**5907.051**	**−6087.69**	**0.807815**	**0.858888**	**0.996457**	**0.092593**	**0.469136**
2	5	−2914.75	5937.492	6539.269	6104.222	6158.222	5831.176	5994.492	5933.27	−6141.51	0.841852	0.86004	0.989887	0.049383	0.450617

Note: Best model is presented in bold. N = 162.

**Table A5:** Panel 2: Number (percentage) of participants assigned to each profile, by diagnostic group, after excluding participants with ID.

Latent Profile/ Diagnostic	ADHD	ID	DLD	LD	ASD	Total
SID	8 (12%)	0 (-)	6 (9%)	1 (8%)	5 (24%)	20 (12%)
SED	16 (25%)	0 (-)	27 (42%)	4 (31%)	4 (19%)	51 (31%)
MED	31 (48%)	0 (-)	27 (42%)	7 (54%)	11 (52%)	76 (47%)
AEP	9 (14%)	0 (-)	4 (6%)	1 (8%)	1 (5%)	15 (9%)
Total	64 (100%)	0 (-)	64 (100%)	13 (100%)	21 (100%)	162 (100%)
